# Special Issue “Molecular Insights into the Role of Exercise in Disease and Health”

**DOI:** 10.3390/ijms26072954

**Published:** 2025-03-25

**Authors:** Brisamar Estébanez, María J. Cuevas

**Affiliations:** Institute of Biomedicine (IBIOMED), University of León, 24071 León, Spain; b.estebanez@unileon.es

Regular physical activity is a fundamental determinant of overall health and a key factor in mitigating the risk of numerous pathological conditions [1,2,3,4,5]. In recent years, considerable research endeavors have been focused on elucidating the molecular mechanisms through which exercise, including mind–body practices, exerts its protective effects on health maintenance and disease prevention [6,7,8,9,10,11]. The present Special Issue aims to provide a comprehensive synthesis of recent advances in the field, offering novel insights into the molecular pathways that mediate the health benefits of exercise and their implications for disease prevention and therapeutic strategies.

In their study, Cantón-Suárez et al. [12] present an exhaustive and contemporary review of the impact of physical activity on Alzheimer’s disease. The paper meticulously examines the effect of diverse physical exercise regimens on the molecular mechanisms underlying the pathophysiological processes that are altered during the development and progression of the disease, utilizing both animal models and human subjects. These mechanisms encompass amyloid β-peptide pathology, tau pathology, neuroglial changes, mitochondrial dysfunction, and oxidative stress. The extant research suggests that physical exercise exerts a protective effect against Alzheimer’s disease due to positive alterations in certain biomarkers linked to the disease’s underlying pathophysiological processes.

Brain-derived neurotrophic factor (BDNF) has also been the focus of extensive study due to its significant physiological role in cell survival and synaptic regulation within the central nervous system [13]. A recent review has demonstrated that high-intensity interval training (HIIT) holds considerable potential as a therapeutic intervention for increasing BDNF levels, with promising outcomes for enhancing brain health and cognitive function [14]. In this regard, the study by Duderstadt et al. [15] is noteworthy for its investigation into the effects of physical inactivity on BDNF levels, particularly in conjunction with controlled normobaric hypoxia. The findings indicated that while physical inactivity resulted in a substantial decline in BDNF levels, exposure to normobaric hypoxia did not trigger a further decrease in BDNF, suggesting that normobaric hypoxia might serve as a counteragent against the drop in BDNF induced by physical inactivity. This underscores the significance of sustaining a physically active lifestyle to ensure neuronal health and lends support to the hypothesis that controlled hypoxia may offer benefits in preventing the decrease in BDNF associated with a paucity of exercise. However, hypoxia can lead to energy crisis, cell death, and tissue dysfunction and promote pathological development [16], such as inflammatory conditions and cognitive impairment [17,18]. Therefore, to establish safe and effective guidelines for the use of controlled hypoxia as a therapeutic intervention, hypoxia protocols must be carefully considered, and further research is essential.

On the other hand, individuals with complete spinal cord injuries exhibit symptoms such as muscular atrophy and an altered metabolic state, a consequence of the conversion of their muscle fibers to a glycolytic and insulin-resistant phenotype [19]. Petrie et al. [20] investigated the potential of electrical muscle stimulation—3 Hz for 1 h vs. 1 Hz for 3 h—to induce beneficial molecular responses in individuals with complete spinal cord injury. The results indicated that stimulation at 3 Hz triggered in the vastus lateralis muscle a substantial activation of early genes (*NR4A3*, *PGC-1α*, *ABRA*, *IRS2*, *EGR1*, *ANKRD1*, and *MYC*) that regulate pivotal processes such as mitochondrial biogenesis (increased number and function of mitochondria), muscle protein synthesis, and metabolic signaling. Given the absence of significant alterations in DNA methylation observed in both protocols, it can be concluded that stimulation-induced changes in muscle function primarily occur through the transcriptional activation of genes, rather than through immediate epigenetic modifications. Thus, electrical stimulation could be an effective strategy to induce beneficial molecular adaptations in paralyzed muscle, and its repeated long-term application could improve muscle metabolism and contractile function.

A body of research has emerged on the molecular mechanisms of physical exercise, including the potential role of small non-coding RNA molecules and extracellular vesicles as intercellular communication and signaling molecules [21]. Wang et al. [22] analyzed how continuous aerobic training and HIIT regulate miRNAs and protein profiles in human plasma-derived extracellular vesicles. The study shows that both types of exercise affect processes such as neuronal signal transduction, autophagy, and cell regulation, albeit with specific differences. Continuous aerobic training relates more to cognitive function and substrate metabolism, whereas HIIT impacts organ growth, cardiac function, and insulin signaling. A particularly noteworthy finding was the identification of distinct sources of extracellular vesicles, including metabolic tissues and the immune and nervous systems, suggesting promising avenues for applications in the prevention and treatment of metabolic, neurodegenerative, and cardiovascular diseases.

These metabolic imbalances may underlie cardiovascular complications [23]. The investigation conducted by Nicolau et al. [24] examined the impact of a supervised exercise program spanning 12 to 14 weeks on high-density lipoprotein (HDL) functionality in patients who had experienced a myocardial infarction. Although a significant increase in the transfer of esterified and non-esterified cholesterol to HDL at the end of follow-up was observed in the total population, no significant improvements in HDL functionality were found. It is particularly noteworthy insofar as it concerns its implications for the transfer of cholesterol to HDL—a process that has been associated with the primary prevention of cardiovascular events [25,26]. Thus, the research of Nicolau et al. [24] underscores the necessity of a more profound understanding of the ways in which exercise programs affect the biological functions of patients with heart disease. Such findings have the potential to guide future research, with the aim of optimizing therapeutic and post-infarction rehabilitation approaches.

Menopause has been shown to be a risk factor for cardiovascular disease in women, and cardiovascular changes occurring during perimenopause may further increase the risk of cardiovascular disease [27]. In their investigation, Wang et al. [28] sought to ascertain the effects of two forms of exercise, namely moderate-intensity continuous training and HIIT, on the bone microstructure and cardiovascular risk factors of ovariectomized female mice. The results of the study indicated that both forms of exercise led to enhancements in blood lipid profiles and vascular structure, accompanied by a reduction in systolic blood pressure and uncarboxylated osteocalcin levels. However, moderate-intensity continuous training emerged as a more effective modality in enhancing bone mineral density and facilitating recovery of bone microstructure. This study contributes to the existing body of knowledge concerning the potential benefits of exercise in mitigating cardiovascular and bone-related complications associated with estrogen loss in postmenopausal women. It sheds light on the therapeutic benefits of physical activity in this particular biological stage and has the potential to influence future exercise recommendations aimed at preventing or treating these conditions in women.

Exercise has also been shown to affect metabolic responses in conditions such as type 2 diabetes (T2D) [29], a condition characterized by muscle metabolic dysfunction [30]. At the molecular level, Garneau et al. [31] investigated myokine secretion in response to a 10-week aerobic exercise program in patients with T2D, showing no significant differences in serum and culture media-derived myokine biomarkers from subjects’ myotubes pre- and post-intervention and in response to an in vitro model of muscle contraction. However, the investigation revealed the potential involvement of certain myokines, including IL-1β, IL-8, IL-10, and IL-15, in exercise endurance among individuals with T2D. While the results were not conclusive, the study highlights the significance of exploring the molecular underpinnings of exercise endurance in diabetes and the necessity to further investigate the potential influence of myokines on metabolic adaptations to exercise in individuals with T2D. This enhanced understanding could pave the way for more personalized and effective therapeutic interventions in the future.

Furthermore, T2D has been identified as a risk factor for the development of left ventricular diastolic dysfunction and heart failure with preserved ejection fraction [32]. Guan et al. [33] conducted a study to analyze the potential mitigating effects of endurance exercise on diabetes-induced diastolic dysfunction in a mouse model. Their findings suggest that exercise promotes cardiac mitophagy, the process of removing damaged mitochondria through autophagy, via the phosphorylation of Ulk1 at the S555 site. Following a period of six weeks involving voluntary wheel exercise, diabetic mice exhibited enhancements in endurance capacity, diastolic function, myocardial oxidative stress, and mitochondrial structure and function. Notably, these benefits persisted even in mice genetically modified (using CRISPR/Cas9) to block this phosphorylation at Ulk1 (S555A), indicating that the improvement in diabetes-induced diastolic dysfunction after exercise is not contingent on Ulk1 phosphorylation at S555. Consequently, the study by Guan et al. [33] offers novel insights into the mechanisms by which endurance exercise combats diastolic dysfunction in diabetes, suggesting that, although Ulk1-mediated mitophagy plays a significant role, there are other factors contributing to the benefits of exercise. This discovery could be pivotal in the development of more effective treatments for cardiac complications in individuals with diabetes.

Metabolic alterations have been demonstrated to affect cellular function and inflammation. Within the framework of ulcerative colitis, a disruption of metabolism is observed, which is associated with changes in the intestinal barrier, immune response, and microbiome [34]. Jin et al. [35] evaluated the combined effect of treadmill exercise and 5-aminosalicylic acid treatment in a mouse model of ulcerative colitis. They observed that the combination of both treatments improved histological damage, reduced inflammation and apoptosis, and promoted colon recovery. At the molecular level, these benefits appear to be mediated by the inhibition of the activation of the NF-κB/MAPK signaling, suggesting that exercise could enhance the therapeutic effects of 5-aminosalicylic acid and offer a new strategy to treat ulcerative colitis.

Finally, Yoon et al. [36] investigated the effects of long-term endurance exercise on inflammatory responses and endoplasmic reticulum stress in the colon, liver, and gut microbiome. The findings indicated that exercise contributes to maintaining intestinal integrity by increasing the expression of crucial epithelial barrier proteins (MUC2, Occludin, and Claudin-2) and activating cellular stress response pathways, including BiP-mediated and eIF2α phosphorylation. Moreover, the investigation identified a correlation between microbial diversity and BiP expression, suggesting a potential role of exercise in influencing the composition of the microbiome as well as metabolic processes, including fatty acid metabolism.

The studies presented in this Special Issue demonstrate the pivotal function of exercise as a therapeutic modality in a variety of neurological and metabolic disorders, owing to its capacity to regulate diverse molecular mechanisms. The evidence derived from these studies reinforces the necessity for an integrated approach, wherein exercise emerges as a promising avenue for the management of various chronic diseases and the enhancement of overall well-being.
